# Thermal Performance of Micro Hotplates with Novel Shapes Based on Single-Layer SiO_2_ Suspended Film

**DOI:** 10.3390/mi9100514

**Published:** 2018-10-11

**Authors:** Qi Liu, Guifu Ding, Yipin Wang, Jinyuan Yao

**Affiliations:** National Key Laboratory of Science and Technology on Micro/Nano Fabrication, School of Electronic Information and Electrical Engineering, Shanghai Jiao Tong University, Shanghai 200240, China; Liuqi94@sjtu.edu.cn (Q.L.); yipinwang@sjtu.edu.cn (Y.W.); jyyao@sjtu.edu.cn (J.Y.)

**Keywords:** suspended micro hotplate, single-layer SiO_2_, temperature uniformity, power consumption, infrared image

## Abstract

In this paper, two kinds of suspended micro hotplate with novel shapes of multibeam structure and reticular structure are designed. These designs have a reliable mechanical strength, so they can be designed and fabricated on single-layer SiO_2_ suspended film through a simplified process. Single-layer suspended film helps to reduce power consumption. Based on the new film shapes, different resistance heaters with various widths and thicknesses are designed. Then, the temperature uniformity and power consumption of different micro hotplates are compared to study the effect of these variables and obtain the one with the optimal thermal performance. We report the simulations of temperature uniformity and give the corresponding infrared images in measurement. The experimental temperature differences are larger than those of the simulation. Experimental results show that the lowest power consumption and the minimum temperature difference are 43 mW and 50 °C, respectively, when the highest temperature on the suspended platform (240 × 240 μm^2^) is 450 °C. Compared to the traditional four-beam micro hotplate, temperature non-uniformity is reduced by about 30–50%.

## 1. Introduction

A micro hotplate (MHP), a heater isolated on a membrane, can be used in gas sensors, micro heat meters, gas flow meters, infrared light sources, and so on [1,2,3]. It presents a range of advantages, such as a miniaturized size, low power consumption, fast thermal response, high sensitivity, compatibility with CMOS technology, ease of integration with other microelectronic devices, and so on [4].

**Figure 1:**
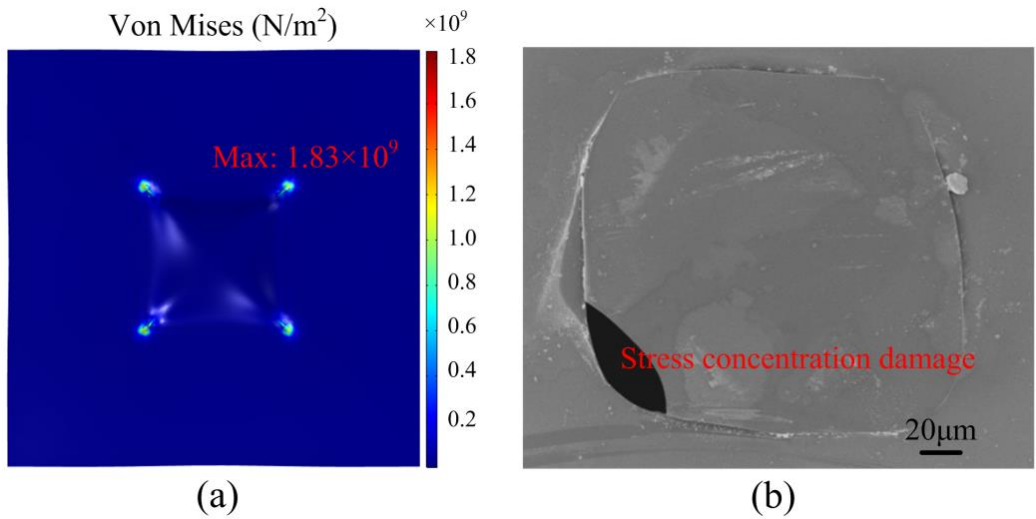
Closed-membrane type structure micro hotplate: (**a**) Stress distribution; (**b**) SEM image of SiO_2_ film.

There are two types of micro hotplate structures: The closed-membrane type, and the suspended-membrane type [5,6]. The closed-membrane type has a faster heat dissipation, and more power loss under the same temperature [7]. The closed-membrane type structure generally requires multilayer stacking to balance the excessive internal stress attained after backside etching. We designed a closed-membrane type micro hotplate with only a layer of 2 μm SiO_2_; the simulation results are shown in Figure 1. The maximum stress on 2 μm SiO_2_ film obtained by backside etching was 1.83 GPa (compressive stress), which was far beyond the stable residual stress limit of 0.1 GPa [8]. Therefore, a closed-membrane type structure with single-layer SiO_2_ can easily be damaged during fabrication, as shown in Figure 1b. To balance stress, many of the previously designed micro hotplates use multilayer composite films, because SiO_2_ has compressive stress, while Si_3_N_4_ has tensile stress. S.Z. Ali designed a micro hotplate including passivation, a metal heat spreading plate, a tungsten heater and silicon heat spreading plate in silicon dioxide, buried silicon dioxide, and substrate from top to bottom [9]. G. Saxena came up with a micro hotplate containing insulation nitride, a heater, an Si_3_N_4_/SiO_2_ composite membrane, and substrate [10]. I. Simon presented a design with passivation, metal heater and electrodes, and a dielectric layer of SiO_2_ and Si_3_N_4_ [11]. For low stress Si_3_N_4_, the closed-membrane type micro hotplate can be successfully fabricated; however, due to the large size of the micro hotplate, power consumption is as high as 250 mW [12].

**Figure 2:**
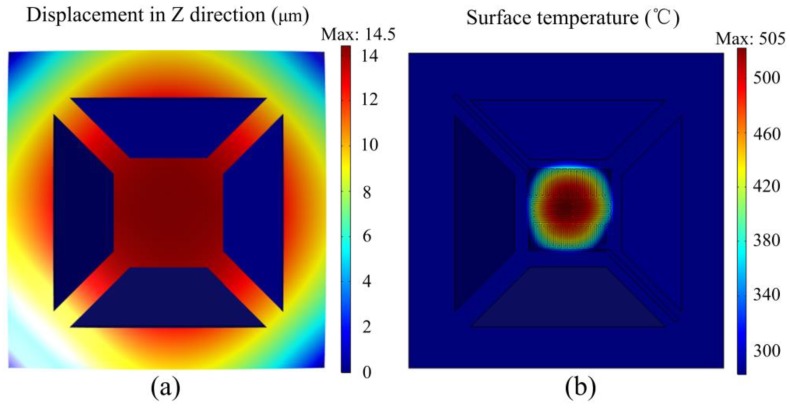
Four-beam suspended structure micro hotplate: (**a**) Deformation; (**b**) Temperature distribution.

The suspended-membrane type micro hotplate has also been widely investigated, but the shape of suspended film is monotonous and there are still problems with the strength and temperature uniformity. F. Samaeifer performed complicated processes of five photolithography with four masks to fabricate the suspended-membrane micro hotplate with Si island [13]. M. Kaur developed a double spiral hotplate with non-uniform temperature distribution on the suspended platform and stress concentration at the connection between the beam and the platform [14]. M. Prasad designed a micro hotplate with 120 × 120 μm^2^ SiO_2_ film; the etching depth was only 56 μm, but the temperature difference was more than 100 °C [15]. Suspended films with different numbers of beams were designed in [16], which had the drawback of fragility; the stress on the beams and the vertical displacement of the plate were quite large. We performed simulation calculations of the traditional four-beam structure in Figure 2, and the deformation under residual stress was up to 14.5 μm, while the temperature difference on the platform exceeded 250 °C. A comparison of various micro hotplates is summarized in Table 1. For the purpose of solving stress concentration and non-uniform temperature distribution, novel shapes of suspended SiO_2_ film were designed. Novel shapes of suspended films have been demonstrated in previous paper for their better mechanical properties [17].

In this paper, micro hotplates with novel shapes of single-layer SiO_2_ suspended film (multibeam structure and reticular structure) were designed and fabricated using extremely simplified processing steps. The goal of new designs was to reduce power consumption and improve temperature uniformity, based on a single layer of a suspended supporting membrane with a reliable mechanical performance. The novel structures can work stably with only a layer of SiO_2_. Finite element simulation guides the design of membrane structure and resistance heaters. The thermal characteristics of these new designs were tested and clear infrared temperature distribution images are given to obtain a design with the optimal comprehensive performances.

## 2. Materials and Methods

### 2.1. Fabrication Details

**Figure 3:**
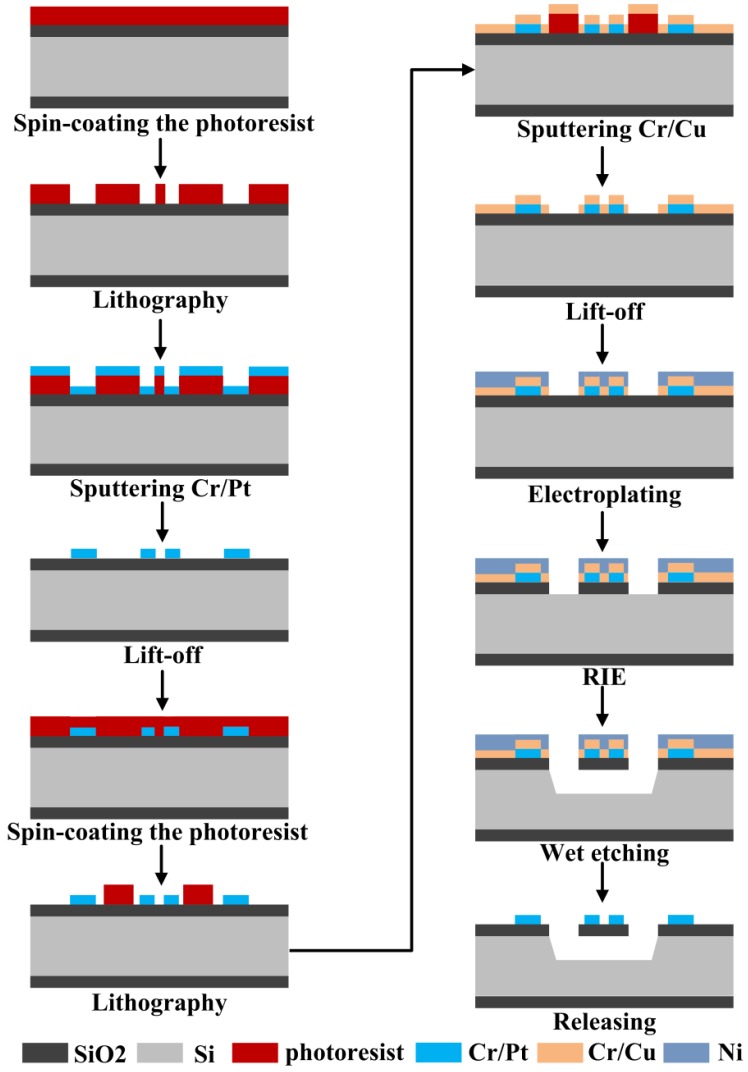
Specific process flow diagram.

The micro hotplates were fabricated on 500 μm double-side oxidized and polished p-type <100> Si wafer; the oxide layer was 2 μm thick. An SiO_2_ membrane was used as a supporting layer and a dielectric membrane to thermally isolate the heated area from the silicon substrate. Only two chrome masks were used to fabricate the micro hotplate: One for resistive heater and electrode, the other for the front-side etching window. A specific process flow, including spin-coating the photoresist, lithography, sputtering, lift-off, electroplating, reactive ion etching (RIE), and wet etching, is shown in Figure 3.

Before spin-coating the photoresist, a tackifier layer of HMDS (Hexamethyldisilizane) was spin-coated and dried on a 150 °C hot plate for 5 min to enhance the adhesion of photoresist and substrate. Otherwise, graphics were prone to fall off during lithography. AZ 4330 photoresist was spin-coated at a speed of 6000 rpm to obtain a thickness of 2.5 μm. The Pt resistance wire and a seed layer of Cr/Cu was deposited by magnetron sputtering. A Ni protective layer of about 2.5 μm was electroplated as a mask for RIE to avoid Cu oxidization; a CHF_3_ and O_2_ gas mixture was used in RIE etching. We used 95 °C 15% KOH solution to perform anisotropic wet etching of the silicon substrate; it took about 2.5 hours to etch about 350 μm, so that the platform could be completely suspended. During the wet etching process, the Ni metal layer effectively protected the front structure and the suspended film. Then, we removed metals Ni, Cu, and Cr to release the device. Finally, the micro hotplate was dipped in ethanol, acetone, and freon (F113) in turn to clean and exchange out water in the cavity. Freon evaporates quickly with low stress, so as to avoid excessive stress and damage in the structure during drying.

### 2.2. Design

Micromachined metal oxide gas sensors with a micro hotplate often need to be able to operate at a temperature which ranges from 200 °C up to 400 °C, and the temperature distribution needs to be as uniform as possible [19]. Metal platinum is used as a heating resistance wire, as platinum has stable physical and chemical properties, and perfect consistency of processing technology. Additionally, it has a large temperature resistance coefficient and a favorable thermal stability, which is extremely suitable for temperature calibration [20]. For the temperature uniformity of the entire platform, the Pt heating resistance wire evenly bestrewed the heating platform. The number, length, and width of the beam linking platform to the base would affect the conduction of heat, so we designed two types of suspended films, a multibeam structure and reticular structure, to study the effect on thermal performance. After that, serpentine and double spiral heating wires were designed, changing the width and the thickness to study the influence of these parameters on the thermal and electric characteristics.

**Figure 4:**
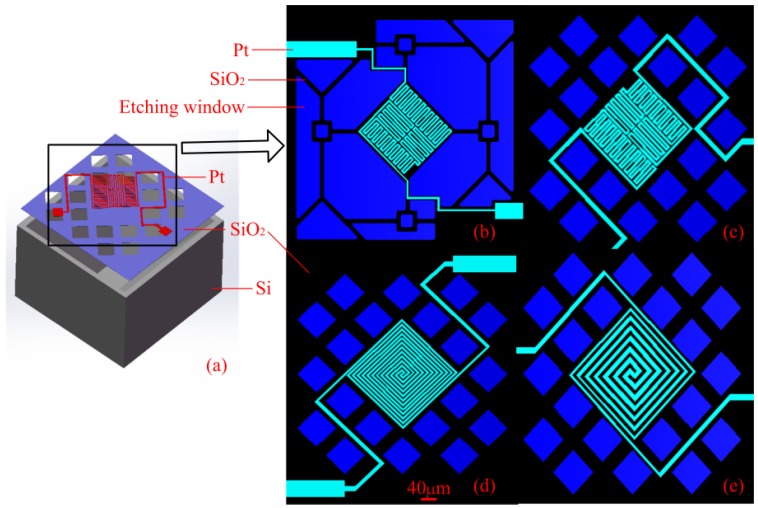
Designs of the micro hotplates: (**a**) Stereogram; (**b**) Design 1: Multibeam structure with a serpentine heater; (**c**) Design 2: Reticular structure with a serpentine heater; (**d**) Design 3: Reticular structure with a double spiral heater; (**e**) Design 4: Reticular structure with a variable line width heater.

A stereogram of the micro hotplate and several combinational designs of suspended films and heaters are shown in Figure 4. At the top surface there was a Pt heating resistance wire; designs 1 and 2 were serpentine heaters with a line width of 5 μm, design 3 was a double spiral heater with 5 μm line width, and design 4 was double a spiral heater with a variable line width of 10 μm, 8 μm, 5 μm from inside to outside. The suspended SiO_2_ membrane consisted of the heating platform and beams connecting between platform and Si base; the cavity was etched in silicon substrate. Design 1 was named a multibeam structure, whereas the other designs were named reticular structures. By KOH anisotropic wet etching, the cavity presented an inverted pyramid with a flat bottom surface. The membrane side length was 720 μm and the heater area side length was about 240 μm. Design 1 and 2 were compared to see the impact of the shape of suspended film; design 2 and 3 were compared for the impact of the Pt wire geometry, and design 3 and 4 were compared for the impact of the Pt wire line width.

### 2.3. Simulation

**Figure 5:**
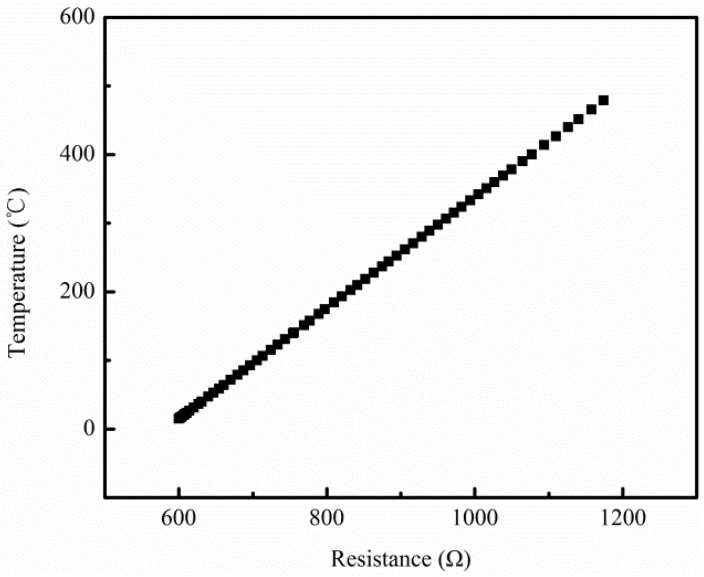
Temperature-resistance calibration curve.

Electrothermal-solid mechanics coupling finite element simulation by Comsol Multiphysics 5.2 was applied to calculate surface temperature distribution of the micro hotplates, by which to compare the above four designs, so as to guide an optimum design with more preferable temperature uniformity. The material parameters used in the simulation are shown in Table 2. The temperature coefficient of resistance (TCR) of Pt was set as 2.063e-3; this value was obtained by the temperature-resistance calibration of sputtered Pt wire. The calibration curve is shown in Figure 5.

**Figure 6:**
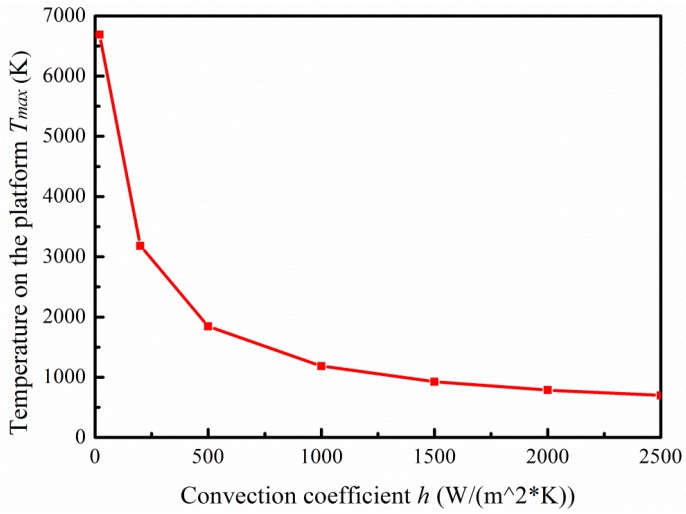
Relationship between convection coefficient *h* and the highest temperature on the platform *T_max_.*

Primary heat loss is the heat conduction of membrane and convection heat dissipation around the environment. Regarding the research on micro hotplates, there were contradictions regarding the main form of heat loss between convection and conduction in the thermal model. The literature [21] pointed out that convective heat loss was the main factor when the temperature was 50 °C higher than ambient temperature. In our simulation, the setting of the convective heat transfer coefficient directly affected the results. The convective heat transfer coefficient is closely related to the shape and size of the heat transfer surface, the temperature difference between the surface and the fluid, and the flow velocity of the fluid. The convection coefficient under the microscale is much higher than the conventional scale [2,22,23]. The cavity inner surface, the heating platform surface, and the heating wire surface were microscale regions with a high temperature, so the convection coefficient (expressed as *h*) was set high, while other surfaces of the base with room temperature were set as an air natural convection coefficient. Take design 1 for example, where the convection coefficient *h* was set as different values to plot the relationship curve between *h* and the highest temperature on the platform *T_max_.* As shown in Figure 6, when *h* is below 1500 W·m^−2^·K^−1^, the value of *T_max_* is greatly affected by the setting of *h*. Thus, it was more reasonable to set *h* between 1500 to 2500 W·m^−2^·K^−1^ to reduce error caused by the choice of *h* value.

**Figure 7:**
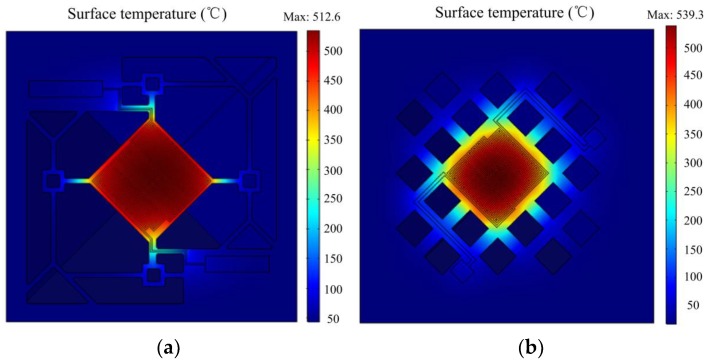

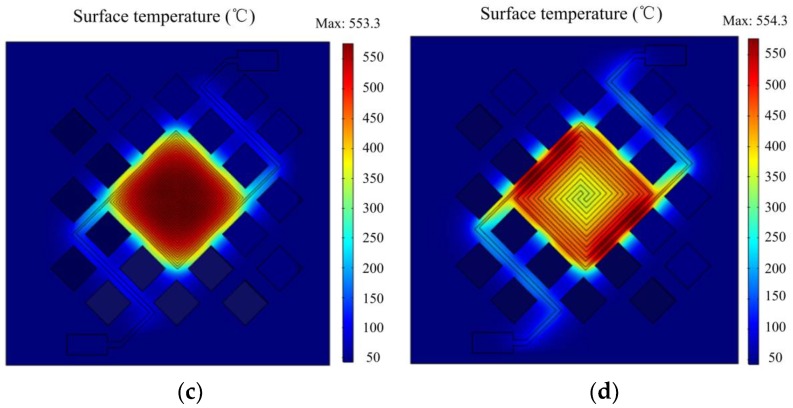
Simulation results: (**a**) Surface temperature of design 1; (**b**) Surface temperature of design 2; (**c**) Surface temperature of design 3; (**d**) Surface temperature of design 4.

When *h* was set to 2000 W·m^−2^·K^−1^, the Pt resistance wire was regarded as a heat source with a DC voltage on electrodes, and the bottom of the base was fixed. Thermal conduction and convection were both taken into consideration; the temperature distributions of the four designs are shown in Figure 7.

Figure 7a indicates that the highest temperature on the platform of design 1 reached 512.6 °C, with a power consumption of 46.8 mW. Temperature decreased gradually outward from the center. The temperature difference on the whole suspended platform was about 120 °C. In addition to the outer ring, temperature difference was within 50 °C in an area of 180 × 180 μm^2^.

As shown in Figure 7b, the highest temperature of design 2 reached 539.3 °C with a power consumption of 61.2 mW. The temperature difference on the whole platform was within 200 °C, while the temperature difference was 100 °C in an area of 180 × 180 μm^2^. Thus, with a similar maximum temperature, the platform surface temperature difference of design 2 was about two times larger than that of design 1. The reticular membrane had thicker, shorter, and more connecting beams, so temperature conduction was stronger than that of the multibeam structure, leading to faster heat dissipation.

Figure 7c indicated that the highest temperature of design 3 was 553.3 °C, with a power consumption of 65.8 mW; at the moment, the temperature in the most central area was around 550 °C. The temperature in a range of 120 μm × μm^2^ was uniformly distributed over 525 °C, and temperature difference in the whole platform was up to 180–200 °C. Comparing with Figure 7b, although the temperature difference on the entire suspended heating platform was almost the same, in the center region of 120 × 120 μm^2^, the temperature difference was less than half of that of the serpentine heater.

It could be seen from Figure 7d that when the highest temperature of the platform reached 554.3 °C, the temperature difference on the whole platform of the variable width heater was the same as that of the identical width heater in Figure 7c. The biggest difference was that temperature on the center of the platform was lower, whereas the outer ring temperature was relatively high because of the finer resistance wire. The temperature uniformity of the above four designs was much better than the that in the four-beam structure shown in Figure 2b, illustrating that novel suspended films had more uniform temperature distribution. Finer and longer beams helped to slow thermal conduction, and the larger etching window led to rapid convection.

**Figure 8:**
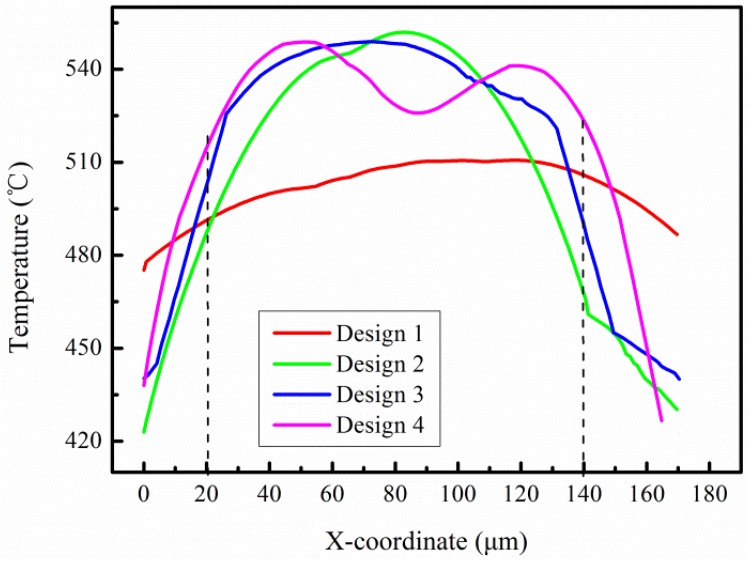
The temperature distribution on the same position line.

For a better comparison of the temperature difference of the four designs, the temperature distribution on the same position line is shown in Figure 8. In the center of a 120 μm range, the temperature difference of the four designs was 20 °C, 80 °C, 60 °C, and 35 °C, respectively. The smallest temperature difference of design 1 shows that thermal conduction through beams has a great influence on temperature uniformity in the simulation.

### 2.4. Experiment

**Figure 9:**
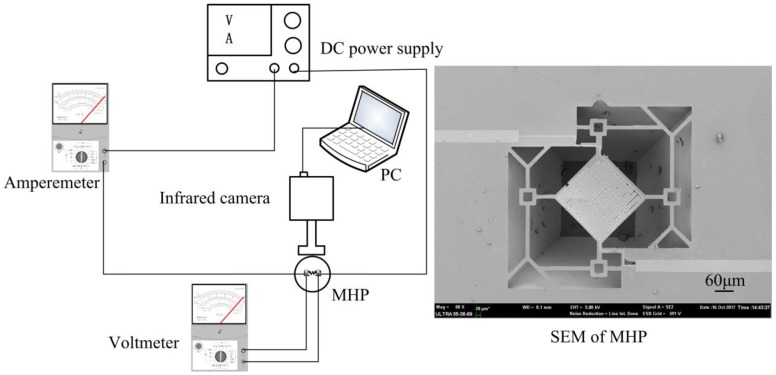
Test system diagram.

The copper wires were soldered on the electrodes and connected to the ammeters through the clamps. In order to facilitate the welding lead, the designs extended the leading wires of the micro hotplates to 4 mm, and expanded the electrodes to 2 × 2 mm^2^. As in the test system diagram shown in Figure 9, DC voltage was applied to the sample, and the heating area temperature distribution was measured using an infrared camera Compix 320, with a pixel size of 6 μm. An amperemeter was used to measure on-state current, while a voltmeter was used to measure the voltage of the sample; thus, different power values were calculated under different DC voltages. Scanning electron microscopy (SEM) of a sample is also shown in Figure 9.

## 3. Results and Discussion

### 3.1. Temperature Uniformity

**Figure 10:**
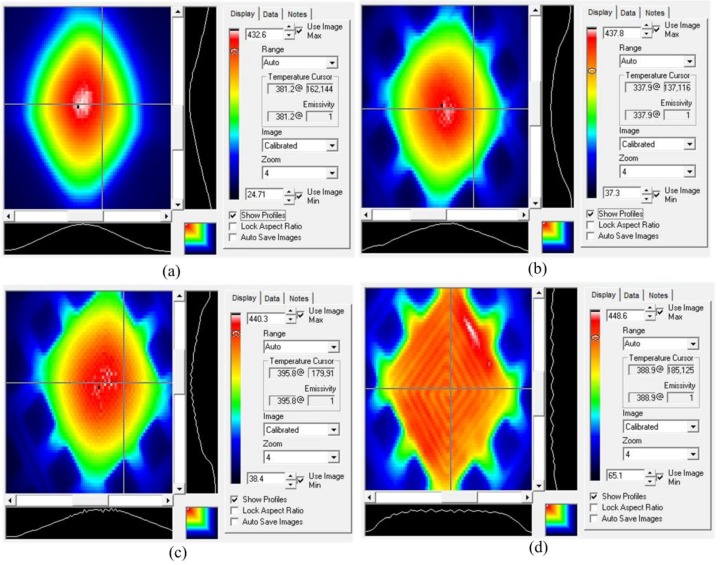
Test results: (**a**) Surface temperature of design 1; (**b**) Surface temperature of design 2; (**c**) Surface temperature of design 3; (**d**) Surface temperature of design 4.

After repeated power-on and power-off for several times, a stable infrared thermogram of design 1 was captured (Figure 10). Applying a voltage of 20 V, at the image capture moment, the highest temperature on the central region was 432.6 °C. Figure 10a shows that the temperature was 381.2 °C at the cross point, and the temperature difference on a quarter of an entire micro hotplate (60 × 60 μm^2^) near the center could be within 50 °C, and in the half area of the whole platform (120 × 120 μm^2^), the temperature difference range was within 90 °C.

The temperature distribution of design 2 under the voltages of 19 V is shown in Figure 10b. The highest temperature on the center was 437.8 °C. The region with a temperature difference of less than 100 °C did not exceed half of the total area of the platform (120 × 120 μm^2^). With a similar highest temperature, the platform surface temperature difference of design 2 was larger than that of design 1, and this was consistent with the simulation. The thermal conduction of the reticular membrane was stronger than that of the multibeam structure, for it had thicker, shorter, and more connecting beams.

Figure 10c shows the temperature distribution of design 3 under the voltages of 20 V, where the highest temperature on the center was 440.3 °C. The regional extents of 50 °C and 100 °C temperature difference were almost the same as those in Figure 10b, so only improving heating wire shape from serpentine to double spiral had little effect on temperature uniformity.

Figure 10d shows the temperature distribution of design 4 under 22 V voltage, where the highest temperature was 448.6 °C and the temperature difference on the whole platform was almost within 50 °C. Compared with that of the traditional four-beam micro hotplate listed in Table 1 [13,15], temperature uniformity was increased by about 30–50%. The temperature profiles swept by two orthogonal straight lines on the same position of four designs are also shown in Figure 10. The temperature profile of design 4 was much flatter than that of others, and showed a more uniform temperature distribution. In actual infrared tests, design 4 has the most uniform temperature distribution, which proves that temperature uniformity can be significantly improved by a reasonable layout of line width. Due to the presence of etching windows and the cavity, the convection at the edge of the platform is stronger than that at the center. The resistance wire with a finer width has high heat to compensate for the strong convection of the edge position.

For designs 1–3, temperature decreased from the center to the edge of the platform, and the temperature difference in the experiment was larger than that in the simulation. For design 4, in the simulation, the temperature of periphery was higher due to the thinner resistance wire, while the temperature of the whole platform was more uniform in the experiment. These phenomena were due to the fact that convective heat dissipation was achieved only by setting a constant convection coefficient *h* in the simulation, but in actual tests, the convection of periphery was more prominent than that of the center because of the existence of the cavity. Previous literature [5,7,10] only had the results of simulations, but infrared temperature images were not given. However, simulation results cannot truly reflect temperature distribution.

### 3.2. Power Consumption

**Figure 11:**
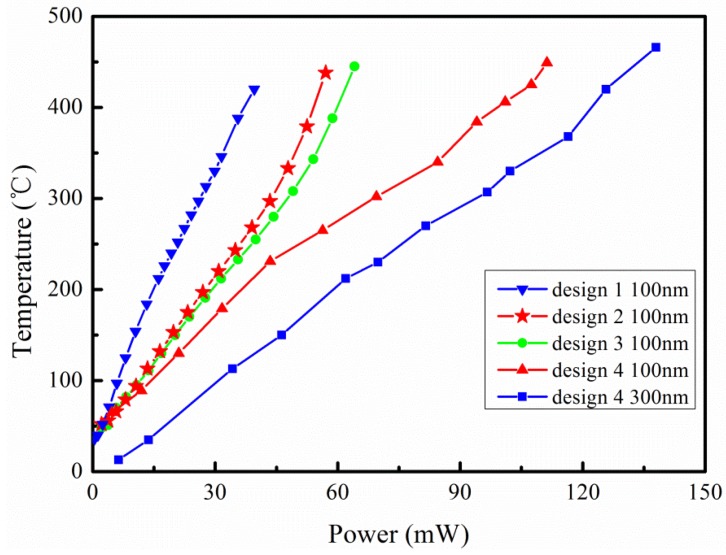
Relationship of power consumption and temperature.

The powers and the corresponding temperatures at different voltages were measured to draw Figure 11. The relationship of the power consumptions on the applied voltages and the average temperatures for the 120 × 120 μm^2^ active area under these conditions was monitored. As the temperature rose, the power was approximately proportional to the increase. For design 1, when average temperature reached 430 °C, power consumption was only 41.32 mW. For design 2, power consumption was 59.10 mW when average temperature reached 430 °C. For design 3, when average temperature reached 430 °C, power consumption was 63.53 mW. For design 4, with the thickness of 100 nm, when the highest temperature reached 445 °C, power consumption was 115.48 mW, with a thickness of 300 nm, and when the highest temperature reached 450 °C, power consumption was 140.01 mW. These values accorded with the fact that micromachined sensors typically require power consumption which is in the range between 30 mW and 150 mW [18].

It could be seen that when the temperature of the platform was almost the same, the power consumption of the reticular membrane structure (design 2) was higher than that of the multibeam structure (design 1). This phenomenon contributed to a larger heat dissipation area and multiple short conduction paths of the reticular membrane structure. The power consumption of the double spiral resistance wire (design 3) was slightly higher than that of the serpentine shape (design 2). Comparing design 4 to design 3, with the same shape of resistance wire, power consumption in the high temperature region increased obviously as line width increased. When the width and the shape of the resistive heaters were the same, power consumption increased sharply with the increase of thickness. In conclusion, the main influencing factors of power consumption are the line width and thickness of the resistance wire.

When heated to 500 °C for about 2–3 min, a 100 nm thick resistance wire was broken due to electromigration. By comparison, when voltage was added to heat up 300 nm thick resistance wire to 650 °C, the micro hotplate could still work. Therefore, resistance wire becomes more reliable when increasing the sputtering thickness; meanwhile, triplicating the section of the wire leads to three times more current before reaching the critical current density for electro migration. Increasing the thickness leads to more power consumption. Thus, an appropriate thickness of the resistance wire needs to be chosen to prevent failure and excessive power consumption.

## 4. Conclusions

Micro hotplates with novel shapes of single-layer SiO_2_ suspended films were designed and fabricated through a simplified technological process. Accurate temperature distribution can be obtained by high resolution infrared images. Finer, longer beams can reduce thermal conduction and small etching windows can reduce heat convection, both leading to a more uniform temperature. The shape change of resistive heaters has little effect on power consumption and thermal uniformity. Wider resistive heaters consume more power, but a reasonable variable line width design compensates for the strong convection of the edge position to improve temperature uniformity on the platform. The heaters should not be too thick, because power consumption increases significantly with increasing thickness. Double-spiral resistance wire with variable, thin line width and moderate thickness based on multibeam structure suspended film is the most optimized design. The lowest power consumption and the minimum temperature difference are 43 mW and 50 °C, respectively, when the highest temperature on the suspended platform (240 × 240 μm^2^) is 450 °C. Compared to that of the suspended micro hotplates listed in Table 1, the temperature difference of this design drops by about 30–50%.

## Figures and Tables

**Table 1:** Comparison of various micro hotplates.

Structure	Membrane size/Heater size (μm^2^)	Maximum Temperature/Temperature Difference (°C)	Power Consumption (mW)	Type	Ref.
SiN/Cr/CrN/Pt/CrN/Cr/	2500 × 2500	498	2350	closed	[1]
SiO/SiN/Glass/SiN	1000 × 910	22		membrane	
Pt/PI film/PI sheet	200 × 200	300	15.5	closed	[2]
	100 × 100	22		membrane	
SiO/Poly-Si/SiN/SiO/	1400 × 1600	460	250	closed	[3]
Si/SiO/SiN	1100 × 1100	90		membrane	
Poly-Si/SiN/SiO/Si/SiO	500 × 500	645	62.8	closed	[7]
	340 × 340	--		membrane	
Insulation nitride/heater/	120 × 120	421	30	Closed	[10]
SiN/SiO/Si	100 × 100	22		membrane	
Pt/SiO/TaN/SiO/SiN/SiO/	300 × 300	450	100	Closed	[18]
Si/ SiO/SiN/SiO	100 × 100	30		membrane	
Pt/SiO/Si/SiO	50 × 50	400	11.8	suspended	[6]
	--	--		membrane	
Pt/SiO/Si/SiO	1000 × 1000	500	50	suspended	[13]
(with Si island)	440 × 440	30(with Si island) >80(without Si island)		membrane	
Pt/SiO/Si/SiO	500 × 500	600	--	Suspended	[14]
	--	>100		membrane	
Pt/SiO/Si/SiO	120 × 120	500	20	suspended	[15]
	--	>100 °C		membrane	

**Table 2:** Material properties in simulation.

Material	Density (Kg/m^3^)	Young’s modulus (Pa)	Poisson’s Ratio	Thermal conductivity (W/m*K)	Thermal-expansion coefficient (1/K)
Si	2330	1.9e11	0.2	148	2.5e-6
SiO_2_	2200	70e9	0.17	1.4	0.5e-6
Pt	21,450	168e9	0.38	71.6	8.8e-6
